# Evidence for Decision-Making: The Importance of Systematic Data Collection as an Essential Component of Responsive Feedback

**DOI:** 10.9745/GHSP-D-22-00246

**Published:** 2023-12-18

**Authors:** Rachel F. McCloud, Mesfin A. Bekalu, Tom Vaughan, Lydia Maranta, Eliz Peck, K. Viswanath

**Affiliations:** a Dana-Farber Cancer Institute, Boston, MA, USA.; b National Institute of Mental Health, Bethesda, MD, USA.; c M&C Saatchi, London, United Kingdom.; d Reckitt, Slough, United Kingdom.; e Hill+Knowlton Strategies, London, United Kingdom.; f Harvard T.H. Chan School of Public Health, Harvard University, Boston, MA, USA.

## Abstract

The authors discuss the importance of systematic data collection as a central component of the responsive feedback process and highlight several case studies that illustrate continuous learning and improvement.

## Key Messages

- Responsive feedback (RF) is based on a philosophy of continuous learning and improvement that relies on the systematic collection of data to generate high-quality evidence that can inform program decisions and changes.
- Data used in an RF approach should be systematically collected through methods that consider the scope and resources available to that program.
- The RF evidence process of continually gathering data to address program assumptions or hypotheses can minimize uncertainty in decision-making.

## INTRODUCTION

The responsive feedback (RF) approach is based on the philosophy of continuous monitoring and learning to improve active programs in real time.^1^ The main characteristics of RF include agility and adaptivity—with the ability to be responsive to lessons learned on where changes or course corrections within a program are needed—and taking action to make these changes, often within iterative cycles.^1^ The capacity and capability to gather data and use evidence is foundational to the RF approach, as it allows for testing of assumptions to determine if changes are needed to increase program success. However, there is often a lack of clarity on the types of evidence that are valuable in an RF approach and how to find a balance between feasibility and perceived rigor of data collection strategies when choosing and implementing methods to collect data to inform programmatic decisions. In this article, we will discuss the main uses of evidence within an RF approach, potential methods for informing RF, and considerations for gathering quality data.

## THE VALUE OF EVIDENCE IN DECISION-MAKING

The use of evidence to make and support programmatic decisions is a key component of the RF process. Dammann^2^ provides a useful description of the path from data that have been collected to the generation of evidence. Data can be defined as the raw numbers, values, and text gathered from research, surveys, interviews, observations, and experiments. Information is considered data in context or data that have been analyzed and/or presented in a way that is meaningful. Finally, evidence is information that has been synthesized in a way that may demonstrate support (or lack thereof) for an argument or set of assumptions and leads one to accept or not accept that argument. Put another way, evidence is information that can be used to test and potentially support a hypothesis. Given the range of program activities or studies that use the RF approach, there may not be a formal hypothesis to test; however, as previously mentioned, RF often centers on testing program assumptions, such as those included in a theory of change, to see if they hold. Because RF relies upon timely hypothesis/assumption testing to make programmatic decisions, it is critical to be mindful of this hierarchy of data, information, and evidence. This will ensure that program staff can chart a clear path from the data gathered to the information derived to the ability to confirm or reject a hypothesis about the program through the evidence. Having this path can streamline activities to prioritize the most relevant data collection strategies that can be used to make real-time programmatic decisions.

Programs that use an RF approach reap 2 benefits of this evidence. First, evidence can lead to a potential confirmation of a hypothesis, such as when a program activity or component in question is performing at optimal levels. Second, evidence can help point to potential solutions to address any issues that have been identified. These suggested modifications could be to the program design, execution, or other features that impact the effectiveness of the intervention. Thus, evidence can help guide the way forward at the following different points within an RF approach.

Evidence can lead to a potential confirmation of a hypothesis or help point to potential solutions to address any issues that have been identified.

1. Evidence can be used to understand the context in which a program will function. Context may refer to several factors that shape the landscape in which the program operates, from the policies that dictate the range of services available to the interpersonal factors that may influence how program content is perceived. The environment in which the program is implemented has an integral influence on program success. At the beginning of a program, assessing relevant factors in the environment—and how they may impact the success of proposed program activities—is a key step to success. Factors such as the larger environment (e.g., regulatory considerations, media influence, and political landscape), community environment (e.g., community resources, locally held beliefs, and local health priorities), and programmatic environment (e.g., program resources such as staff capabilities or time and organizational priorities) may exert influence on whether activities can successfully fully achieve their outcomes. Furthermore, the needs, interests, and barriers of the intended population or audience may influence their receptivity to and ability to act upon program content. Understanding these background factors—particularly those that are relevant to program activities and their implementation—is a useful first step to looking critically at the “workings” of the program and how these factors may operate in this environment.
2. Evidence helps program staff to assess and understand what is working well in their program and what areas may need to be fixed. This often means exploring different sources of evidence to determine if activities are on the right track, such as examining process data to determine if attendance to classes or training sessions is as expected. Attendance numbers that are lower than anticipated may signify the need to collect additional data (such as brief interviews or surveys) to discover why attendance is low. For example, the Minnesota Heart Health Program's campaign to promote health screening in 3 communities learned that fewer people in 1 community were coming to the health center to get screened compared to those in other communities. Researchers examined interim data gathered by the program and realized that the channel used to reach the intended audience in the low-performing community did not have sufficient reach. Specifically, 2 intermediate markers used by the program (brand awareness and name recall) were reported in much lower numbers, signaling that the messages were not reaching the intended audience. During the campaign, the program switched channels from TV to direct mail and increased the number of people visiting the local health center for screening.^3^
3. Evidence can inform potential program decisions or strategies. Programs may contain certain decision points, such as what type of message to use or what strategy to implement. Sometimes, this means choosing between different options to determine which is the optimal action to take to improve the program. RF can be a useful tool to structure brief experiments or comparison tests mid-program. For example, MTV Shuga (Supplement) is a campaign in Nigeria that discusses reproductive health through a popular television drama series and accompanying social media content. Researchers wanted to test different messaging strategies for increasing social media content among the intended program audiences. They used their previously established Facebook page to test different thematic versions of content and used metrics, such as engagements and video views that are automatically gathered by Facebook, to decide which future strategies should be used. Using this iterative approach, they were able to determine the post topics and language that would generate the most views and engagement on their Facebook site.
4. Evidence can be used when collaborating with stakeholders to understand findings and determine potential new directions. The clear reporting of evidence can create a transparent process so others can understand the reasoning behind these proposed changes. Often, it is helpful to discuss these findings as a group with staff and stakeholders to determine potential next steps for program action, as community-based stakeholders and clients can have valuable insights and feedback to add to the process. The RF process also highlights the value of pause-and-reflect sessions—periodic gatherings where diverse stakeholders (e.g., program leadership, monitoring and evaluation staff, program staff, and others who are key to a program's success) convene to provide feedback, and in many cases, help to further understand how to interpret the evidence. Having a succinct story or summary of the data gathered and how it points to the need for programmatic change can be a valuable tool to engage others in the decision-making process so that they may have a starting point for their reactions and feedback. This process allows for multiple viewpoints and interpretations of findings to be considered that may help the team arrive at the best course of action. For example, the Viswanath Lab COVID-19 dashboard (Supplement) used a combination of Google Analytics data, social media engagement data, and frequent conversations with community stakeholders to adapt the content of COVID-19 messages and how they were disseminated. Using this approach, the Lab identified different strategies that could enhance their reach within different communities. Listening to the needs and priorities of community leaders and public health staff led to the creation of messages that were delivered in the style, format, and channel that best suited each audience and the cultural context.

## POTENTIAL METHODS FOR COLLECTING DATA FOR RF

Methods are strategies, processes, or techniques used to gather raw data that can then be combined to generate evidence. These methods create the structure for systematically collecting data to inform program decisions. Systematic data collection occurs when there is a fixed plan or system for data collection, which may mean there is a protocol in place for collecting data from participants (such as through a predetermined set of survey questions or an established interview guide) or a form or guide for gathering observational data. This system underscores the importance of eliciting buy-in from program staff and ensuring that all interviewers, survey administrators, observers, or analysts are trained in the program's aims, overall protocol, and methods. Systematic data collection also ensures that researchers are conscious of potential biases, use well-established principles or rules in gathering the data, and are cognizant of the limitations of the data.

The methods that guide data collection may vary according to the needs of each program. For RF, these methods center on gathering data while the program is still active. As such, methods prioritized in this approach may need to occur in a more condensed time interval or use fewer resources compared to methods used to measure the outcome of an intervention. Furthermore, when selecting a method for RF, it is important to ensure that there are adequate resources to gather, analyze, and interpret data during program implementation. For example, types of data used in documented RF case study examples have included ethnographic observations or quantitative social media data, as with MTV Shuga (Supplement). Other studies have used design workshops, consultations with organizations,^4^ household surveys, telephone surveys, and previously collected program data.^5^

When selecting a method for collecting data for RF, it is important to ensure there are adequate resources to gather, analyze, and interpret data while the program is operating.

There are a variety of well-established methods that may be used to collect data for RF. Primary data collection methods may range from quantitative (e.g., surveys) to qualitative (e.g., focus groups, interviews, ethnographic observations). There are many ways of incorporating primary data collection methods into RF research, even when faced with resource constraints. For example, a brief quantitative survey may gather feedback from participants within a few questions. Key informant interviews may help to gather detailed feedback from a few integral sources when there are no resources to gather data from a large number of respondents. Program staff may also find value in secondary data that have already been captured for other purposes, such as examining past surveys or analyses of social media postings to answer critical programmatic questions. Evidence derived from secondary data can be valuable when time and resources are limited because it does not require additional effort to collect new survey data or interviews. However, it is important to note that when using secondary data, the way the data were gathered may not align directly with what is needed to answer a certain program assumption or question; that is, there may not be an exact fit with the data gathered and the evidence needed to answer a specific question in the current context.

Experiments are often used within RF to rapidly test different potential approaches to advance iterative change. Often, these experiments are constructed to compare 2 variations, iterative pilot tests, or test-and-learn cycles. Although methods, such as randomized controlled trials, are often hailed as the gold standard, they are often difficult to conduct within RF programs due to their increased time and cost demands, as well as the inability to carefully control conditions within the community context. For this reason, experiments that are smaller in scope and resources are prioritized for RF activities.

Process data—either quantitative, qualitative, or both—are often a key component used to evaluate RF programs. These data may be crucial to determine if the program or activity is operating as expected and may highlight areas that need improvement within these programs. It is particularly beneficial to incorporate a plan for gathering process data efficiently into the overall program design, prioritizing methods that can help answer important questions and be quickly analyzed to inform the program's next steps.

Methods used will be determined based on the needs and scope of the program and should be geared toward answering the specific issues addressed by that program. For example, a program in its nascent stages of design may opt for qualitative methods with key informants or community exemplars that can provide more detail on the context in which the program will operate. However, if more information is needed on a wide swath of the population, brief surveys may more effectively reach a wider area of respondents and allow more rapid analysis.

RF methods emphasize gathering data that are collected systematically and clearly documenting how data were gathered and the justifications for using certain methods and measures. Because RF-based data are often reviewed in real time so that decisions can be made quickly, having well-documented data can facilitate this prompt review and will increase the ability to illustrate to others how these findings guided actions.

There may be situations in which more than 1 method can be used to gather data for RF decision-making, such as combining focus groups and surveys to see how many people in a program might be performing a certain behavior (survey) and then try to understand why (focus group). The USAID Takamol program, a gender equity program in Jordan, used several sources of data in different phases of the project to gauge engagement and program acceptance with the intended audience. When quantitative data revealed that youth (an important audience group for the program) were only a small percentage of the community members who participated in program activities, staff used focus groups with youth, adaptation sessions with staff, and market research to determine how to increase engagement with this group. Throughout the process, Takamol staff also held meetings with local government and civil society organizations to reflect on what was—or was not—working and why. Using this approach, they identified several successful strategies to increase engagement and reach among youth.^6^

RF evidence does not always need to arise from introducing a new method or data source. As previously mentioned, it may be beneficial to assess secondary data to determine if they can be used to inform RF activities or to provide additional context that can help to support programmatic decisions. For example, in the SKY Girls preventative tobacco control program, telephone survey responses revealed that there was a low level of exposure to program content and that these surveys were not able to reach many girls who had seen program materials. To overcome these limitations and fill knowledge gaps on why this low exposure was occurring, program staff decided to analyze a SKY Girls database that was originally part of a separate project to gather information on teens exposed to project activities. Analysis of this database indicated that Facebook was less effective at delivering messages compared to other sources. Based on the additional context provided by this evidence, program staff shifted their approach away from Facebook for future activities. Subsequent rounds of mobile phone surveys were administered to determine if this shift was successful and indicated a steady increase in exposure to the campaign and a positive perception of the project brand.^5^

## CONSIDERATIONS WHEN USING EVIDENCE IN RF

### Evidence Quality

A key goal of the RF evidence process is to use evidence to proceed more confidently with programmatic decisions and actions. In other words, it may not be possible to gain absolute certainty about the correctness of decisions, but continuing to gather data using the RF approach to address program assumptions or hypotheses can minimize uncertainty. Data collection should be structured in a way that is systematic and clear and reduces bias. The goal is to gather high-quality data regardless of the selected method(s) that can allow more confidence in the results found from this process.

Given the broad scope of potential sources of RF evidence, there is no 1 marker for evidence quality. However, decades of research have shown there are some core principles and tenets that serve as criteria to assess data quality (Box).^7^

BOX Criteria for Assessing Quality of Data

Some criteria worth considering include making sure that the data are:

**Valid.** Validity ensures the accuracy of the data gathered—that they are truly measuring what they intended to measure.

**Reliable.** Reliability refers to whether the measures produce stable and consistent results—that a measure captures information in the same way for different populations or different participants. For research involving observations, it means that those recording the observations would report or code information in the same way (2 different observers report the same observation).

**Representative.** It is important to understand how data do or do not include the range of voices that may have feedback to offer, even if it may not be timely or possible to include all viewpoints. This may require careful consideration of the identification of the sample (intended audience of the program) who will be providing the data. Furthermore, it is useful to determine if the data available are more likely to represent 1 incident or the experience of 1 person as opposed to an experience common to many.

**Credibly Sourced.** When considering a data source, it is valuable to know where the data came from and that it is considered a source of accurate information. Sources that may be of particular value are those with prior knowledge of the subject or program, such as program staff, leaders of the community, or program participants.

Given the realities of the program context, the options for gathering data may be limited or bound by certain time or resource constraints, particularly during times of crisis. However, these criteria may still serve as useful guidelines for program staff to put in place to strategically use limited time and resources. These criteria may (1) help guide strategy for collecting the best quality data possible and (2) help program staff critically evaluate the quality of the data they have gathered as they use it to test hypotheses and form conclusions or plan next steps. For example, if data are needed during an emergency to help rapidly iterate a program that is operating in an unstable and quickly changing political climate, program staff can prioritize their work to ensure that data are uniformly gathered and thus easier to analyze. Even if programs have the capacity to quickly gather only feedback from staff, this feedback can be gathered strategically and systematically to address the program assumptions in question.

Programmatic realities must also be considered when determining the level of representation that evidence can achieve. For example, time constraints and geography must be considered when selecting the sampling strategy that may be used when conducting surveys within a community. Using the criteria to assess quality allows program staff to take a critical view of what assumptions they may make or initial conclusions they may draw from considering this completeness or lack thereof.

Similarly, examining the credibility of sources used can be a valuable tool to more quickly generate high-quality evidence. Before data are gathered, program staff may determine if the sources that are providing these data are credible and are adding value to the program. Citing that these data came from a credible source may also increase the value of findings for donors or funders. Program workers directly involved in implementation often serve as a key resource in providing on-the-ground insights into how the program is operating in reality. Finding ways to quickly and systematically collect these insights can help to rapidly identify potential areas that are not working as expected in a program and focus efforts to gather data to determine the best ways to pivot. Having a trusting relationship and open communication channels with donors can amplify this process.

The Le Wi Ol Lan (LWOL) project was a small-scale program operating in Sierra Leone to address challenges in the education system and improve in-school learning opportunities and outcomes. When the Ebola outbreak hit Sierra Leone in 2014, quick and sizeable changes were needed to school-based programs, such as LWOL, as they could no longer operate in their original form once schools closed with no reopening date in sight. Soon after schools closed, LWOL conducted a risk assessment of the outbreak's impact on learning. Evidence gathered from this process indicated the need to shift to an alternative model focused on smaller group learning. As the outbreak progressed, LWOL donors encouraged an iterative project design that created opportunities to pilot new and innovative approaches with rapid assessments to determine if these new approaches were successful. An essential factor contributing to the LWOL project's success in this crisis was the established, trusting relationship with the donors that included channels for documenting feedback from project implementing staff. Another was the reliance on feedback from those directly involved with program implementation because their combined observations could quickly generate evidence on whether new program directions were worth pursuing. As these observations were gathered, the donors empowered the teams to systematically pilot and learn from these new ideas. The success of this approach directly inspired the Improving School in Sierra Leone program that supports 450 schools across 8 districts. This case highlights the importance of clear communication with donors in an iterative, flexible environment. Another school-based program in Sierra Leone that had a more rigid program structure was shuttered for months during this time.^8^

### Rigor and RF

Scientific rigor refers to the execution of a study design that is detailed enough to be repeated, is free from bias, and contains complete and accurate reporting that clearly states the rationale for design decisions. There are situations when evidence is required to be a product of the highest standards of rigor, whereas, in other situations, less rigorous methods will do as well. Tightly controlled conditions under which an intervention takes place help to reduce bias, the conscious or unconscious influencing of a study and its results. Everyone has biases from their own cognitions and experiences, and these thoughts may tend to be generalized as program decisions are made. Rigor helps protect from those biases. Some study designs are structured such that they have a higher level of rigor (e.g., experiments with randomized control and intervention groups) (see Potential Methods).

Rigor is an important scientific concept and provides a valuable framework for establishing cause and effect between variables. However, the most traditionally rigorous designs may not always be conducive to the conditions faced during program implementation. For example, those working to address a humanitarian crisis may not have the time and resources to conduct an RCT, or those seeking a way to structure their pilot study may want a method that provides more rich detail in their formative research with participants. As we discussed in the Potential Methods section, the nature of the program, its goals, resources, and research questions may influence the methods and study designs that may be used.^9^ Regardless of the nature of the program, it is still possible to structure, gather, and document evidence in a way that increases confidence in the conclusions that are drawn. A critical step to reducing bias is to create a documented trail from evidence to decisions to actions that can promote collective reflection.

A critical step to reducing bias is to create a documented trail from evidence to decisions to actions that can promote collective reflection.

The final decision on whether to implement a change to a program will rely on several factors, including both the magnitude of the change and features of the programmatic environment in which the change would be implemented. Given these factors, there may be different requirements for the quality or level of rigor of evidence that may be needed to justify a decision. At the end of this process, organizations or teams may determine if the evidence has provided the level of confidence needed to proceed with a program change or if more evidence is needed before making a decision.

## DISCUSSION

Considering the importance of strategically gathered data in on-the-ground programs is a promising way to build agility into program design and execution. Conceptualizing a path from raw data into quality evidence can streamline data gathering to pinpoint areas for improvement. Even when programs implement data collection after initial design and inception, having a clear plan to systematically gather data in a way that allows for it to be used to answer key questions is of utmost importance to foster the ability to pivot quickly during implementation. The RF approach allows for program staff to take a holistic view of the program, its stakeholder priorities, and the context in which the program operates. Through this lens, it can provide guidance on how to be strategic and systematic with how evidence is generated and used. Furthermore, the RF approach values the input of several types of evidence and highlights the value of contributions from many different methods. Even when there are extreme time constraints, following these criteria can allow program staff to prioritize gathering quality data as is feasible given the programmatic context and provide an additional understanding of what questions the available evidence can and cannot answer. This indicates that many different methods may be used in low-resource settings around the world and provides parameters for data gathered to be a part of evidence generation. As we illustrate in this article, having a clear and succinct way to contextualize and make meaning of evidence and findings to donors and other key stakeholders in a way that links to their priorities can also be a valuable tool to turn evidence into action.

## CONCLUSION

In summary, evidence can serve as a guidepost to informing programs, strengthening the ability to make confident decisions, and allowing transparency in the process of making those decisions with stakeholders. In light of these benefits, the authors advocate for an RF decision-making process that does the following.

- Considers the scope of the program and its resources to implement feasible evidence-gathering strategies
- Draws from the appropriate methods that can systematically capture evidence
- Relies on quality evidence that provides clear information

## Supplementary Material

GHSP-D-22-00246-supplement.pdf

## Notes

**Peer Reviewed**

**First published online:** September 25, 2023.
