# Supplementary material for: Evidence for Decision-Making: The Importance of Systematic Data Collection as an Essential Component of Responsive Feedback
Source: Glob Health Sci Pract. 2023 Dec 18;11(Suppl 2):e2200246. doi: 10.9745/GHSP-D-22-00246 (PMC10727464; doi:10.9745/GHSP-D-22-00246)
Supplement: GHSP-D-22-00246-supplement.pdf [file GHSP-D-22-00246-supplement.pdf]

Supplement to: McCloud RF, Bekalu MA, Vaughan T, Maranta L, Peck E, Viswanath K. Evidence for decision-making: the importance of systematic data collection as an essential component of responsive feedback. *Glob Health Sci Pract*. 2023;11(Suppl 2):e2200246. https://doi.org/10.9745/GHSP-D-22-00246

SUPPLEMENT. RESPONSIVE FEEDBACK CASE STUDIES

MTV Shuga

MTV Shuga is a popular television and social media program in Nigeria that focuses on family planning and sexual health. The program wanted to test messaging strategies to see which strategy has the biggest impact in increasing family planning communication and behaviors with their target audience. While the program has a well-established format for their television show, researchers identified their Facebook page as a promising place for testing and iteration.

They realized that they did not know the best ways to construct messages on this platform that would work for their broad target audience. Key questions emerged about what message themes and formats (such as language) may attract the most attention and would garner the most likes or shares.

The goal was to create new messages and then test them to determine their performance among Facebook followers of MTV Shuga through a series of test and learn cycles (that is, a series of planned experiment cycles within a program that test strategies iteratively and modify program content based on the findings). Researchers wanted to use methods that would help them to first determine what message theme and language received the most engagement with viewers. The first iteration of the test focused on 2 potential themes identified from formative research ("sex myths" or "talk about it") by providing a post and related video on each topic, each in 4 languages.

Researchers leveraged the vast amounts of social media data that were already being collected from the Facebook platform as their evidence for message engagement. For the first phase, they used the metrics of engagements, video views, and average watch times to see which message performed the best. These were measures that are already widely used and vetted by Facebook to gather quality data. The use of social media data also allowed for the potential inclusion of a wide range of audience members' engagement behaviors within their evidence.

The English version of the "talk about it" video resonated with the widest audience—1.3 million viewers. This pointed clearly that the next phase of testing should focus on this topic and language to develop messages further in the next cycle—with English content that featured characters from the show talking to their partners about the use of protection.

Supplement to: McCloud RF, Bekalu MA, Vaughan T, Maranta L, Peck E, Viswanath K. Evidence for decision-making: the importance of systematic data collection as an essential component of responsive feedback. *Glob Health Sci Pract*. 2023;11(Suppl 2):e2200246. https://doi.org/10.9745/GHSP-D-22-00246

Viswanath Lab COVID-19 Dashboard

The Viswanath Lab COVID-19 Dashboard was formed in direct response to perceived informational needs during the COVID-19 pandemic. Within the fraught and often conflicting information environment of COVID-19, particularly in early 2020, the Viswanath Lab sought to create a source for evidence-based information on topics such as general information about disease spread and symptoms; prevention through non-pharmaceutical interventions such as masking and social distancing; and ways to address issues such as mental well-being and tobacco use during the pandemic. Vaccine information was added as the pandemic progressed. The dashboard was created on several lab websites with different audiences: a dashboard on the main lab page was intended for a broad, primarily US-based audience, with a particular emphasis on partners in low-income areas of the state. Another dashboard centered on a primarily India-based audience. Each dashboard had sections for key areas of information such as frequently asked questions (FAQs) and myth busters, which addressed commonly circulated pandemic-related myths.

The dashboards were initially created with the purpose of distilling evidence-based information in a format that would amplify important messages in an approachable format. After the first initial push of information onto lab websites, the lab began to examine the initial assumptions of the effort: that people would willingly seek COVID-19 information and would visit the site to gather it. However, suggestions from community partners suggested that might not be the case.

Given the rapid nature of the pandemic and the need for immediate information, the Lab identified methods that could be implemented quickly and efficiently to measure audience engagement. First, Google Analytics were used to determine audience engagement, including number of visits to the site, location of visitors (e.g., country, city), and top pages visited. However, the lab realized that only part of the story could be gleaned from these metrics. The Lab worked closely with community contacts to determine the needs of the community as well.

The sources of lab evidence were valid, reliable, and leveraged the viewpoints of many voices. Google Analytics data provides useful, instant metrics that give a view of the users of the site. However, since this can only capture the characteristics of site usage—not the details of those that did not engage with the site. Staff and community representatives were then able to provide a more representative picture of the needs of the community members. These trusted voices indicated that many of their community members were not likely to visit the website directly. This was supported by low engagement found in the Google Analytics data. Furthermore, community members preferred more visual graphics instead of text in an FAQ section.

This led the Lab to challenge the initial underlying assumption of “if you build it they will come.” Instead, the Lab then focused on creating infographics and GIFs to share on social media and through community-based dissemination channels and organizations.
